# Attenuated dynamic impulse control in risky action under the escalating risk and reward in gambling disorder

**DOI:** 10.1017/S0033291726104322

**Published:** 2026-05-11

**Authors:** Gangliang Zhong, Tianzhen Chen, Jingyang Liu, Yicheng Wei, Xiyuan Zhang, Peiqiong Yang, Ru-Yuan Zhang, Valerie Voon, Jiang Du, Min Zhao

**Affiliations:** 1 Shanghai Mental Health Center, Shanghai Jiao Tong University School of Medicine, Shanghai, 200030, China; 2 School of Psychology, Shanghai Jiao Tong University, Shanghai, 200030, China; 3 Institute of Science and Technology for Brain-Inspired Intelligence, MOE Frontiers Center for Brain Science, Fudan University, Shanghai, 200433, China; 4 Dept. of Psychology and Behavioural and Clinical Neuroscience Institute, University of Cambridge, Cambridge, CB2 3EB, UK

**Keywords:** gambling disorder, impulse control, impulse inhibition, risk evaluation, trait impulsivity

## Abstract

**Background:**

Gambling disorder (GD) involves persistent risky choices despite losses, suggesting impaired impulse control. While static paradigms reveal inhibition deficits in GD, they cannot model dynamic risk-reward escalations during real gambling. This study aims to investigate whether GD involves impaired dynamic impulse control during escalating stakes and to dissociate contributions of subjective risk evaluation and trait impulsivity to this deficit.

**Methods:**

Using a sequential gambling task with 83 male patients with GD and 62 matched healthy controls (HCs), this study investigated dynamic impulse control deficits under escalating stakes. We quantified dynamic impulse control via the reward–reaction time (RT) coupling for ‘continue’ choices (dynamic impulse control index [DICI]) using Bayesian modeling. Risk sensitivity and risk preference were derived from stop/continue decisions. Trait impulsivity was assessed with the Barratt Impulsiveness Scale (BIS-11). Regression analyses examined the modulation of DICI by risk sensitivity and trait impulsivity.

**Results:**

Patients with GD exhibited significantly attenuated DICI versus HCs, reflecting failure to increase deliberation with escalating stakes. Computational modeling revealed markedly reduced risk sensitivity in GD despite comparable risk preference. Critically, trait impulsivity positively modulated DICI in HCs but not in GD, indicating pathological decoupling. Risk sensitivity positively predicted DICI in both groups, though significantly weaker in GD.

**Conclusions:**

These findings establish a triadic impairment in GD: (1) attenuated adaptive impulse control during escalation (impaired DICI), (2) deficient subjective risk weighting (reduced sensitivity), and (3) breakdown of impulsivity-based modulation of control. This reveals a dynamic, mechanism-focused pathology beyond static trait models.

## Introduction

Gambling disorder (GD), recognized as the first formal behavioral addiction in the *Diagnostic and Statistical Manual of Mental Disorders*, Fifth Edition (DSM-5) (American Psychiatric Association, 2013), has emerged as a pressing global public health concern. It is characterized by dysfunctional impulse regulation, wherein individuals continue making risky choices despite incurring escalating financial losses, reflecting a core impairment in behavioral inhibition (Holmes, 2015; Potenza, 2015; Romanczuk-Seiferth, van den Brink, & Goudriaan, 2014). While conventional static paradigms (e.g. stop-signal tasks, Stroop paradigm) reliably capture basic response inhibition deficits in GD (Gavazzi et al., 2025; Kräplin et al., 2014; Potenza et al., 2003), they fail to simulate the dynamically escalating risk-reward contingencies central to real-world gambling scenarios, which demand continuous inhibitory control. Crucially, it remains unexplored whether GD involves a specific breakdown in the dynamic impulse control mechanisms necessary for navigating such increasing stakes, a defining feature of pathological gambling progression.

In neurotypical individuals, contexts involving escalating risks and rewards engage an adaptive cortico-subcortical ‘braking’ network, including regions like the pre-supplementary motor area and inferior frontal gyrus, facilitating a shift toward more rational decision-making as potential stakes rise (Meder et al., 2016). Activation within this network scales with the magnitude of potential gains and losses, underscoring its role in context-sensitive behavioral adjustment (Meder et al., 2016). Conversely, patients with GD exhibit functional abnormalities in key nodes of this regulatory circuitry, such as the ventromedial prefrontal cortex and orbitofrontal cortex (Clark, Lawrence, Astley-Jones, & Gray, 2009; Gavazzi et al., 2025; Kaasinen et al., 2023; Potenza et al., 2003; Sescousse, Barbalat, Domenech, & Dreher, 2013), which contribute to context-driven risk biases that cannot be fully explained by trait impulsivity models alone (Fujimoto et al., 2017; Power, Goodyear, & Crockford, 2012). These findings collectively highlight impaired impulse regulation during evolving risk contexts in GD; however, the specific behavioral and computational processes governing dynamic impulse control under conditions of escalating financial stakes remain uncharacterized.

Real-world gambling often involves a sequence of decisions where individuals repeatedly choose to ‘continue’ gambling, accepting progressively higher risks, or to ‘stop’ to secure accumulated gains, a dynamic process that demands ongoing evaluation of changing contingencies. A diagnostic feature of GD is ‘loss chasing’, persistent gambling to recoup losses, and previous research has explored its behavioral and neural underpinnings (Banerjee, Chen, Clark, & Noël, 2023; Campbell-Meiklejohn, Woolrich, Passingham, & Rogers, 2008). For instance, Campbell-Meiklejohn et al. (2008) found that loss-chasing decisions correlate with increased activity in incentive-motivation brain regions, while quitting is linked to anterior cingulate cortex activity associated with conflict monitoring. Complementary pharmacological studies show that modulating catecholamine systems (e.g. via methylphenidate) alters the inhibitory impact of high stakes on persistent risky choices in healthy adults, highlighting the neurochemical sensitivity of these decision-making processes (Campbell-Meiklejohn et al., 2012). Beyond trait risk preferences, Fujimoto et al. (2017) further demonstrated that GD involves deficits in state-dependent risk attitude modulation, linked to diminished dorsolateral prefrontal cortex function, underscoring impairments in flexible behavioral control critical for adaptive gambling.

Notably, while existing research has advanced understanding of GD-related decision deficits, few studies have focused on dynamic within-trial adjustments to escalating stakes, a core feature of real-world gambling. To address this gap, the sequential gambling task (Meder et al., 2016) was employed, as it effectively emulates natural gambling trajectories: participants accumulate rewards through successive ‘continue’ choices, with the risk of substantial loss escalating over time. In healthy individuals, the deliberation time for ‘continue’ choices typically lengthens as cumulative rewards increase, signaling heightened cognitive engagement and inhibitory effort; shortened reaction times under high stakes, by contrast, indicate compromised impulse control (Meder et al., 2016; Smith, Mattick, Jamadar, & Iredale, 2014). This coupling between reaction time and reward magnitude serves as a behavioral index of dynamic impulse control (DICI). More importantly, while other reaction time adaptations like post-error slowing (Penolazzi et al., 2020; Weidacker et al., 2021) or post-reinforcement pauses (Thompson & Corr, 2013) have been studied in GD, the DICI specifically captures the dynamic, within-trial adjustment of deliberation time in direct response to escalating stake magnitudes, thus offering a more precise measure of the inhibitory processes challenged during escalating risk contexts central to pathological gambling.

Furthermore, computational modeling of choices in this task allows estimation of latent subjective factors, such as risk sensitivity, the subjective weighting of escalating stakes, and risk preference, a baseline propensity for risk-taking (Lohse, Løkkegaard, Siebner, & Meder, 2023; Meder et al., 2016). Although GD is associated with elevated trait impulsivity (Ioannidis et al., 2019; Kräplin et al., 2014; Zhong et al., 2024), which undermines sound decision-making (Yücel et al., 2019; Zhong et al., 2025), it is unclear whether the disorder also involves distorted subjective evaluation of escalating risks (e.g. reduced risk sensitivity) and how such evaluation interacts with dynamic impulse control and trait-based impulsivity.

Building upon these gaps, we hypothesized that, compared to healthy controls, individuals with GD would demonstrate (1) attenuated dynamic impulse control, reflected in weakened coupling between reward escalation and reaction times during ‘continue’ decisions; (2) reduced risk sensitivity in evaluating escalating stakes, distinct from risk preference; and (3) impulsivity-mediated deficits in dynamic control, further modulated by abnormal risk sensitivity. To test these hypotheses, we employed the sequential gambling task (Meder et al., 2016) combined with computational modeling (Bayesian trial-by-trial analysis; Fontanesi, Shenhav, & Gluth, 2022) to derive a DICI, risk sensitivity, and risk preference in GD patients and matched controls. Our results confirmed significantly attenuated dynamic impulse control in GD, comparable risk preference relative to controls, and markedly reduced risk sensitivity. Importantly, trait impulsivity positively modulated dynamic control in healthy individuals, an adaptive relationship absent in GD, while risk sensitivity’s moderating effect on control was substantially weaker in the patient group.

## Methods

### Participants

The study initially recruited 95 male outpatients or inpatients diagnosed with GD from the Shanghai Mental Health Center, China, alongside 75 male HCs who did not differ significantly from the GD group in age or education level. GD diagnoses were independently verified by addiction-specialized certified psychiatrists via the Chinese-adapted SCID-5-CV (First, Williams, Karg, & Spitzer, 2020) (see Supplementary Methods). Participants were aged 19–65 years and had no comorbid psychiatric diagnoses within the preceding 6 months, except for GD within the patient group. Following task-based exclusion criteria established by Fontanesi et al. (2022), we excluded participants exhibiting task non-comprehension, defined as either <5% ‘continue’ or ‘stop’ decisions, or premature stopping patterns. Consequently, 12 participants were excluded from the GD group and four from the HC group. Additionally, nine further HCs were excluded after scoring >5 on the Chinese South Oaks Gambling Screen (Zhou et al., 2025), indicating potential pathological gambling. The final analytical sample thus comprised 83 participants with GD (81% online gambling, 19% offline; 21 inpatients: 1.2 ± 0.4 months residential therapy; see Supplementary Methods) and 62 HCs. Among the included HCs, SOGS scores ranged from 0 to 4, with a mean of 0.11 ± 0.48, confirming that gambling involvement was minimal and non-pathological.

### Experimental task and procedures

**Figure 1.**
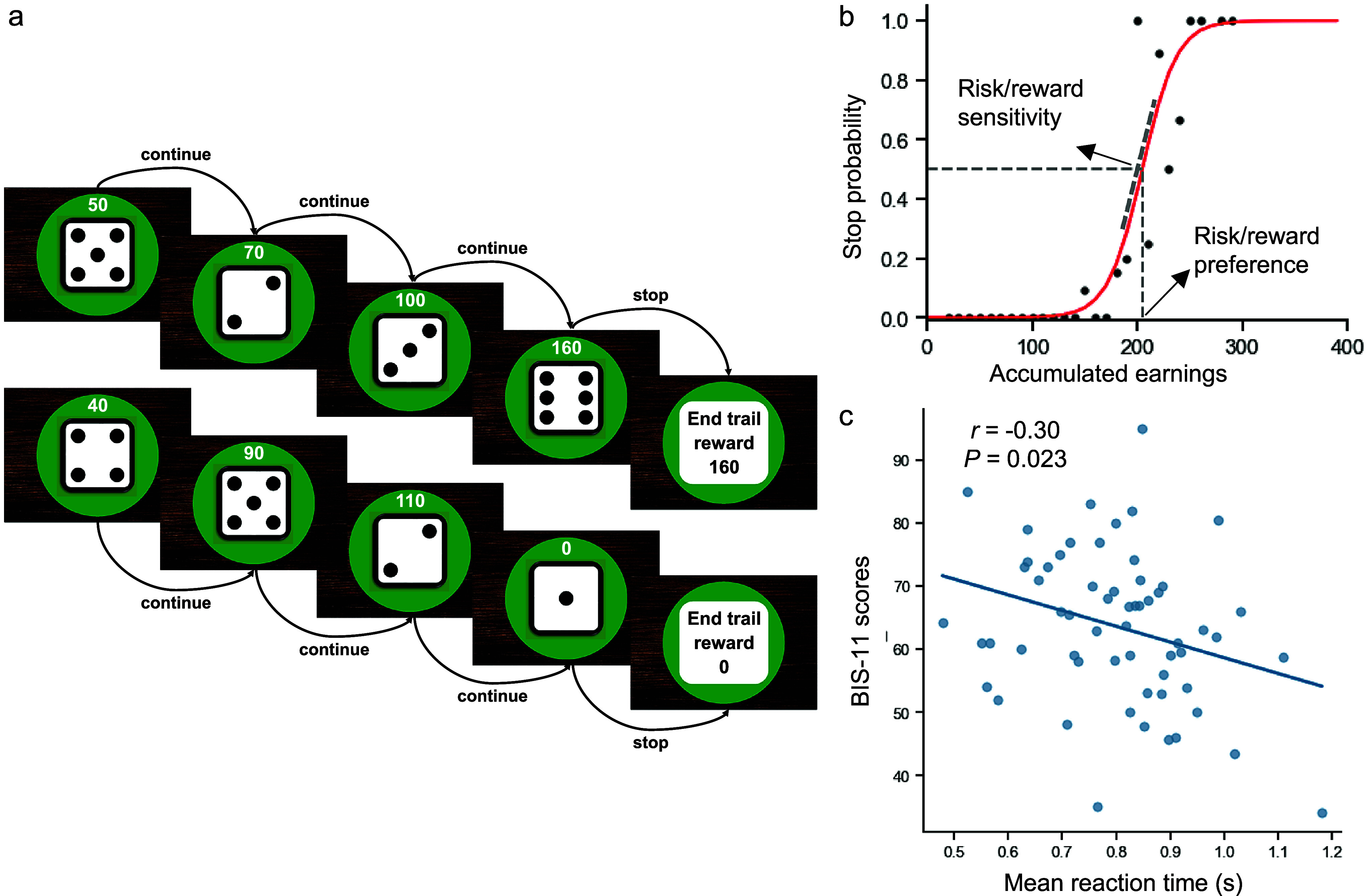
Experimental paradigm, stop probability modeling, and behavior–impulsivity associations. (a) Example of sequential decision-making: Round termination via voluntary stop (securing 160 reward) versus forced termination upon rolling ‘1’ (loss of accrued rewards). (b) Logistic regression modeling individual-level stop probability functions against cumulative rewards (Meder et al., 2016). (c) Association between mean reaction time for ‘continue’ decisions and trait impulsivity (BIS-11 scores) in healthy controls. Abbreviations: BIS-11, Barratt Impulsiveness Scale version 11.

All participants performed the sequential gambling task, originally designed by Meder et al. (2016) and later adopted in relevant studies (Lohse et al., 2023). The task structure (illustrated in Figure 1a) dynamically simulates escalating reward–risk contingencies characteristic of naturalistic gambling contexts. Administered for a fixed 25-minute duration, the total number of completed rounds varied across participants depending on their decision-making speed and choices. On average, participants with GD completed 248 rounds (SD = 15.2), while HCs completed 245 rounds (SD = 14.8), with no significant group difference (*t*(143) = 1.21, *P* = 0.23). Across rounds, participants repeatedly rolled a six-sided die to accumulate monetary rewards, with each subsequent roll carrying a progressively heightened risk of loss. A key feature was sequential binary choices within each round: continue gambling (risking accrued rewards for potential incremental gains) or stop (securing the round’s cumulative sum to proceed). Each die roll yielded a random outcome (1–6) with equal probability (1/6 per face). Rolling a ‘1’ constituted a loss, terminating the round and forfeiting all accumulated rewards; this event was random and independent of prior rolls, making losses unpredictable to participants at any point. In contrast, rolls of 2–6 led to multiplicative reward increases (value × 10 Renminbi, ∼1.4 USD or 1.3 EUR), after which participants deliberated their next choice.

The task’s escalating risk dynamic arose inherently from the fixed 1/6 per-roll loss probability combined with dynamically growing cumulative stakes. Each successful ‘continue’ choice (i.e. a roll of 2–6) increased the round’s cumulative reward, but the objective loss probability (rolling a ‘1’) remained constant at 1/6. This created a scenario where the potential consequences of a loss intensified with each successive roll, even as the objective odds of loss did not shift. The varying starting amounts and increments for different trial types (illustrated in Figure 1) stemmed from the random sequence of die outcomes (2–6), which introduced variability in reward trajectories across rounds. Although ‘within-round draw number’ and ‘cumulative reward’ were correlated, they were not perfectly collinear due to this randomness, with the same cumulative reward achievable at different draw numbers and vice versa. This design feature afforded sufficient independence to model their distinct effects on reaction time within our hierarchical Bayesian regression framework.

The task does not feature a simple, always-effective strategy to maximize gains because outcomes remain probabilistic. However, based on the fixed probability of loss (1/6 per roll) and the increasing stakes, the theoretically optimal stopping point, where the expected value of an additional roll turns negative, occurs when accumulated earnings reach 200 Renminbi. This is the point at which the potential loss (current earnings × 1/6) begins to outweigh the expected gain (mean reward of 4 × 10 Renminbi × 5/6). Nonetheless, even adhering to this rule does not assure maximum gains in any single round, as avoiding a loss on subsequent rolls remains probabilistic. Thus, the task captures the core dynamic of real-world gambling, where players must weigh escalating risks against potential rewards without a deterministic ‘winning’ formula.

Each round initiated with an automated animated die roll lasting 1.5–3.5 seconds (jittered). Following outcome presentation (2.5 seconds), participants actively indicated their decision within a 2-second response window: pressing a designated button to continue gambling or withholding a response to stop, thereby banking accumulated rewards. Notably, failure to respond within this window defaulted to a stop decision, ensuring expeditious choices. The task was programmed using PsychoPy (v2023.1.3, www.psychopy.org; [Peirce, 2007]).

Before task administration, participants received explicit instructions regarding performance-contingent compensation: in addition to a base payment (50 Renminbi per 30 minutes), they earned bonuses based on the mean accumulated rewards across all rounds, including rounds ending in total loss. All participants were given standardized instructions and completed a brief practice session prior to the formal task. Following the task, trait impulsivity was quantified using the Chinese version of the Barratt Impulsiveness Scale, 11th edition (BIS-11) (Li et al., 2011).

### Dynamic impulse control index quantification

To evaluate behavioral differences in gambling inhibition between participants with GD and HC, we employed a trial-by-trial analysis within the sequential gambling task. In this paradigm, participants continually balanced the escalating risk of losing accumulated rewards (upon rolling a 1) against the disadvantage of prematurely terminating a round for suboptimal gains. Crucially, participants faced repeated decisions to either continue gambling amid dynamically increasing stakes or stop to secure accrued rewards. Convergent evidence indicates that rational decision-makers calibrate behavioral inhibition proportional to escalating stakes (Lohse et al., 2023; Meder et al., 2016). Within this framework, cessation of gambling represents the voluntary inhibition of continued risky actions. Consequently, inhibition propensity is expected to increase monotonically with cumulative rewards and risk escalation (Fontanesi et al., 2022; Meder et al., 2016). Critically, a significant positive linear relationship between accumulated earnings and RTs for ‘continue’ choices preceding voluntary stopping reflects heightened inhibitory engagement, whereas a negative slope suggests diminishing impulse control (Meder et al., 2016). Therefore, we operationalized dynamic impulse control via a trial-by-trial analysis relating accumulated earnings to RTs during ‘continue’ trials preceding active cessation. Specifically, we employed publicly available code (Fontanesi et al., 2022) to perform hierarchical Bayesian linear regressions modeling log(RT) against predictor variables: round number, within-round draw number (sequential dice roll count), and cumulative reward sum. This approach was adopted because these predictors collectively influence RT dynamics, as established by Fontanesi and colleagues.

Crucially, the hierarchical Bayesian linear regression model was specified and estimated separately for the GD and HC groups. The models were run separately for each group to allow for distinct parameter distributions without assuming a common prior, thus facilitating direct group comparisons. Moreover, the individual parameter estimates from these separate group-level models were then used for subsequent regression analyses investigating the modulatory effects of risk sensitivity and BIS scores.

### Risk preference and risk sensitivity measures

On each trial prior to a loss event, participants chose between continuing or stopping gambling, with accumulated rewards offering increasingly strong evidence to cease. Previous work has demonstrated a sigmoidal relationship (Meder et al., 2016), where stop probability increases nonlinearly with cumulative rewards (Figure 1b). Following established methodology (Fontanesi et al., 2022), we estimated individual stop probability functions via hierarchical Bayesian logistic regression, modeling the binary choice (stop vs. continue) as a function of per-round cumulative reward.

Two key parameters, risk sensitivity and risk preference, were derived from these fitted functions. Risk sensitivity is quantified by the logistic regression coefficient for cumulative reward: a higher coefficient indicates greater sensitivity to risk–reward escalation per unit stake change, reflecting a stronger tendency to shift from gambling to cessation under equivalent risk dynamics. Formally, risk sensitivity corresponds to the coefficient (w₁) in the trial-by-trial hierarchical Bayesian logistic regression model: *p*(stop | xₙ) = 1 / (1 + exp(−w₁xₙ − w₀)), where xₙ denotes cumulative reward on trial *n* and w0 and w1 are free parameters. This model captures how stop probability changes with each unit increase in cumulative reward, independent of total stop decisions.

Risk preference is operationalized as the certainty equivalent, the accumulated reward value at which the stop probability equals 50%. This inflection point represents indifference between continuing and stopping. Participants with lower certainty equivalent values terminated gambling earlier, exhibiting lower risk tolerance, whereas higher certainty equivalent values indicated delayed cessation and elevated risk-seeking. Crucially, the models were run separately for each group to allow for distinct parameter distributions without assuming a common prior, thus facilitating direct group comparisons.

### Computational modeling implementation

For both the linear (DICI) and logistic (risk parameters) regression models, Bayesian hierarchical estimation was performed using PyStan. Linear regressions utilized 2 chains with 2000 iterations (50% warm-up). Logistic regressions employed 4 chains with 8000 iterations (87.5% warm-up). Model convergence was rigorously validated by ensuring: (1) R-hat < 1.05, (2) <1% divergent transitions, and (3) <1% iterations reaching the maximum tree depth of 10. The full specifications of hierarchical Bayesian models were provided in Supplementary Table S1.

### Statistical analyses

Analyses were conducted in Python 3.8.18 using SciPy.Stats. Normality was assessed via Shapiro–Wilk tests. Group differences (GD vs. HC) for continuous variables were tested using independent *t*-tests (normal data) or Mann–Whitney *U* tests (non-normal data). Correlations employed Pearson’s *r* (normal data) or Spearman’s ρ (non-normal data).

To test hypothesized group modulations, we conducted two distinct hierarchical regression analyses on participant-level data: (1) Analysis 1 examined effects of group, risk sensitivity (z-scored), and their interaction on DICI; (2) Analysis 2 examined effects of group, trait impulsivity (BIS-11 scores; *z*-scored), and their interaction on DICI. Group (GD/HC) was coded categorically using effects coding. Significant interactions underwent post-hoc decomposition to quantify simple effects.

### Ethics

This study was conducted following the Declaration of Helsinki and approved by the Shanghai Mental Health Center Ethics Board (Protocol #2022-18). All participants provided written informed consent before participation and received monetary compensation.

## Results

### Demographic and clinical characteristics

The HC and GD groups were comparable in age (*U* = 3030, *P* = 0.07, *r* = 0.177) and education (*U* = 2783, *P* = 0.40, *r* = 0.541). Trait impulsivity, assessed using the BIS-11, was significantly elevated in patients with GD (90.64 ± 13.83) compared to HCs (64.03 ± 12.20) (*t*(143) = 12.044, *P* < 0.001, *d* = 2.022), where higher scores denote greater impulsivity. For comprehensive demographics and clinical data, see Table 1a.

**Table 1.** Demographic and clinical characteristics, performance on the sequential gambling task, and hierarchical Bayesian model parameters

Variable	GD (*n* = 83)	HC (*n* = 62)	Statistics
**a. Demographic and clinical characteristics**			
Age (years)	29.00	30.50	*U* = 3030, *P* = 0.07, *r* = 0.177
	(26.00, 32.00)	(26.00, 36.00)	
Education (years)	15.00	15.00	*U* = 2783, *P* = 0.40, *r* = 0.541
	(15.00, 16.00)	(11.25, 18.00)	
BIS-11 (scores)	90.64 ± 13.83	64.03 ± 12.20	*t*(143) = 12.044, ***P* < 0.001**, *d* = 2.022
**b. Performance on the sequential gambling task**			
Mean accumulated earnings (RMB)	131.52	122.28	*U* = 2855*, P* = 0.26, *r* = 0.035
	(103.27, 178.07)	(98.98, 167.46)	
Mean reaction time (s)	0.79 ± 0.17	0.80 ± 0.14	*t*(138) = −0.120, *P* = 0.90, *d* = −0.021
Voluntarily continued decision (*n*)	166.00	155.00	*U* = 2852*, P* = 0.27, *r* = 0.037
	(146.50, 189.00)	(135.00, 181.00)	
Voluntarily stopped decision (*n*)	41.00	50.00	*U* = 2188, *P* = 0.12, *r* = 0.294
	(29.00, 53.00)	(31.50, 61.00)	
Risk-induced loss event (*n*)	41.39 ± 6.64	39.86 ± 4.67	*t*(142.54) = 1.630, *P* = 0.11, *d* = 0.260

*Note*: Continuous data conforming to normality assumptions were summarized using the mean ± standard deviation, with group differences assessed using independent-samples *t*-tests. For data violating the normality assumption, the median and interquartile range are presented, and group differences were evaluated using Mann–Whitney *U* tests. Bold indicates significant group differences (*P* < 0.05). Abbreviations: GD, gambling disorders; HC, healthy controls; BIS-11, Barratt Impulsiveness Scale version 11; years, years; RMB, Renminbi; s, seconds; *n*, count. *d*, Cohen’s d (for *t*-tests); *r*, rank-biserial correlation (for Mann–Whitney *U* tests).

### Choice behavior

In the sequential gambling task (Figure 1a), participants with GD made an average of 166 voluntarily continued decisions, 41 voluntarily stopped decisions, and experienced 41.39 risk-induced loss events. HCs made 155 voluntarily continued decisions, 50 voluntarily stopped decisions, and experienced 39.86 risk-induced loss events. For voluntarily stopped decisions, the mean accumulated earnings were 131.52 Renminbi for GD and 122.28 Renminbi for HCs. For voluntarily continued decisions, the mean reaction time was 0.79 s for GD and 0.80 s for HCs. No group differences emerged in mean accumulated earnings (*U* = 2855*, P* = 0.26, *r* = 0.035), mean RT (*t*(138) = −0.120, *P* = 0.90, *d* = −0.021), number of voluntarily continued decision (*U* = 2852*, P* = 0.27, *r* = 0.037), number of voluntarily stopped decision (*U* = 2188, *P* = 0.12, *r* = 0.294), or risk-induced loss events (*t*(142.54) = 1.630, *P* = 0.11, *d* = 0.260) (see Table 1b for full behavioral data).

To replicate prior findings linking risky choices to trait impulsivity in HCs (Lohse et al., 2023), we operationalized RT during deliberation preceding risky choices (continue decisions) as a behavioral metric of impulsivity. Within HCs, mean RT for continue decisions correlated negatively with trait impulsivity (*r* = −0.3, *P* = 0.023; Figure 1c). This aligns with Lohse et al.’s observation that higher BIS scores predicted larger mean stopping amounts and confirms that behavioral impulsivity (reflected in shortened deliberation time) covaries with trait impulsivity in escalating risk contexts. Thus, RT dynamics during risk/reward escalation effectively index dynamic impulse control underlying continued gambling.

### Attenuated dynamic impulse control in GD

Consistent with prior methodology (Meder et al., 2016), HCs exhibited increased hesitancy to continue gambling as cumulative rewards escalated during sequential trials, reflected in prolonged RTs for ‘continue’ choices. This RT-reward coupling serves as a behavioral index of DICI. To quantify individual DICI, we fitted a hierarchical Bayesian linear regression model (details provided in the Methods section) predicting log(RT) from predictor variables (cumulative reward sum, round number, and within-round draw number). The model demonstrated strong explanatory power for both groups: Bayesian R-squared (Gelman, Goodrich, Gabry, & Vehtari, 2019) median was 0.600 (HDI = [0.592, 0.608]) for HCs (Supplementary Figure S1a) and 0.603 (HDI = [0.597, 0.610]) for patients with GD (Supplementary Figure S1b), aligning with validation benchmarks from Fontanesi et al. (median R-squared = 0.62) (Fontanesi et al., 2022).

**Figure 2.**
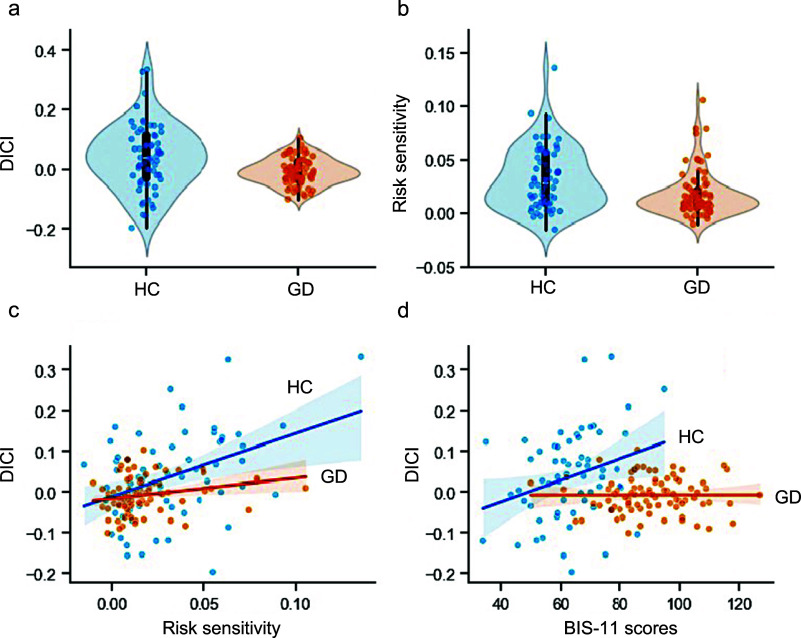
Attenuated dynamic impulse control in gambling disorder reveals blunted modulation by risk sensitivity and trait impulsivity. (a) Significantly attenuated dynamic impulse control index (DICI) in gambling disorder (GD) patients (red) compared to healthy controls (HC, blue; *t*(75.27) = −3.174, *P* = 0.002, *d* = -0.593). Violin plots show probability density distributions with individual data points. (b) Markedly reduced risk sensitivity in GD (red) relative to HC (blue; *U* = 1743, *P* < 0.001, *r* = 0.467). Violin plots and scatter points display distribution characteristics. (c) Differential modulation of DICI by risk sensitivity: significant positive association in both groups (HC: *β* = 0.040 ± 0.011, *t*(61) = 3.458, *P* = 0.001; GD: *β* = 0.013 ± 0.006, *t*(82) = 2.205, *P* = 0.030) but significantly weakened slope in GD (*t*(90.57) = 2.083, *P* = 0.040). Linear regression lines characterize group-specific associations. Shaded bands represent 95% confidence intervals. (d) Impaired trait impulsivity (BIS-11)-DICI coupling in GD: significant positive modulation in HC (*β* = 0.050 ± 0.021, *t*(61) = 2.403, *P* = 0.019), absent in GD patients (*β* = 0.0001 ± 0.0065, *t*(82) = 0.009, *P* = 0.993). Linear regression lines characterize group-specific associations. Shaded bands represent 95% confidence intervals. Abbreviations: HC, healthy controls; GD, gambling disorder; DICI, dynamic impulse control index; BIS-11, Barratt Impulsiveness Scale version 11; *d* = Cohen’s d (for *t*-tests); *r* = rank-biserial correlation (for Mann–Whitney *U* tests).

Critically, group comparisons revealed significantly attenuated RT-reward coupling in patients with GD (*β* = −0.007 ± 0.043; Table 2) relative to HCs (*β* = 0.040 ± 0.110; Table 2) (*t*(75.27) = −3.174, *P* = 0.002, *d* = −0.593; Figure 2a).

**Table 2.** Hierarchical Bayesian model parameter estimates

Variable	GD (*n* = 83)	HC (*n* = 62)	Statistics
DICI	−0.007 ± 0.043	0.040 ± 0.110	*t*(75.27) = −3.174, ***P*** = **0.002**, *d* = −0.593
Risk/reward sensitivity	0.010 (0.006, 0.020)	0.028 (0.010, 0.056)	*U* = 1743, ***P* <0.001**, *r* = 0.467
Risk/reward preference	189.336 (101.047, 377.348)	154.642 (91.442, 236.857)	*U* = 2909*, P* =0.1800*, r* = 0.014

*Note*: Continuous data conforming to normality assumptions were summarized using the mean ± standard deviation, with group differences assessed using independent-samples *t*-tests. For data violating the normality assumption, the median and interquartile range are presented, and group differences were evaluated using Mann–Whitney *U* tests. Bold indicates significant group differences (*P* < 0.05). Abbreviations: DICI, dynamic impulse control index; GD, gambling disorders; HC, healthy controls; *d*, Cohen’s d (for *t*-tests); *r*, rank-biserial correlation (for Mann–Whitney *U* tests).

Thus, the attenuated DICI signifies a failure in dynamic impulse control regulation during escalating stakes, rather than inferior task outcomes per se. One-sample *t*-tests confirmed that RT-reward coupling coefficients were significantly positive in HCs (*t*(61) = 2.847, *P* = 0.006), replicating the normative pattern of risk-/risk-/reward-modulated inhibitory engagement (Meder et al., 2016). In contrast, patients with GD showed no significant coupling (*t*(82) = −1.484, *P* = 0.142), demonstrating failure to upregulate impulse control under heightened risk-reward contexts. Notably, a more homogeneous distribution of DICI values within the GD group compared to HCs (independent-samples *t*-tests, GD: −0.007 ± 0.043; HC: 0.040 ± 0.110; Table 2).

### Lower risk sensitivity in GD

To quantify individual risk sensitivity and risk preference, we modeled stop/continue decisions as a function of cumulative reward sum per round using hierarchical Bayesian logistic regression (details provided in the Methods section). Model fit was robust for both groups: Bayesian R-squared (Gelman et al., 2019) median was 0.606 (HDI = [0.600, 0.613]) for HCs (Supplementary Figure S1c) and 0.574 (HDI = [0.568, 0.580]) for patients with GD (Supplementary Figure S1d). These values align with prior validation benchmarks (Meder et al., 2016: median R-squared = 0.47; Fontanesi et al., 2022: median R-squared = 0.62), confirming adequate model fit.

Group comparisons revealed no significant difference in risk preference (*U* = 2909*, P* =0.1800*, r* = 0.014; Table 2). Critically, risk sensitivity was significantly reduced in patients with GD (*U* = 1743, *P* < 0.001, *r* = 0.467; Table 2; Figure 2b), indicating impaired subjective evaluation of escalating risk-reward contingencies despite intact risk tolerance. Notably, a parallel pattern of reduced variability was observed for risk sensitivity in the GD group relative to HCs (Mann–Whitney *U* tests, GD: 0.010 [0.006, 0.020]; HC: 0.028 [0.010, 0.056]; Table 2).

### Risk sensitivity modulates dynamic impulse control

As shown in Table 3a (modulation by risk sensitivity), the baseline model (Model 1: Group-only) significantly predicted DICI (*R* ^2^ = 0.080, *F* = 12.48, *P* < 0.001). Adding risk sensitivity (Model 2) significantly increased explained variance (Δ*R* ^2^ = 0.112, Δ*F* = 4.43, *P* < 0.001), indicating that risk sensitivity accounted for additional variance in DICI beyond Group. Further adding the risk sensitivity × Group interaction (Model 3) significantly improved the model (Δ*R* ^2^ = 0.023, Δ*F* = −3.98, *P* < 0.001), confirming that the interaction explains unique variance in DICI beyond main effects.

**Table 3.** Moderation effects of risk sensitivity and trait impulsivity on dynamic impulse control in gambling disorder and healthy controls

Parameter	*AIC*	*R* ^2^	Δ*R* ^2^	Δ*F*	β (SE)	*t*	*P*
**a. Modulation of DICI by risk sensitivity**							
***Model 1***	−323	0.080	0.080	12.48			**<0.001**
Group (HC or GD)					−0.05 (0.01)	−3.53	0.001
***Model 2***	−340	0.192	0.112	4.43			**<0.001**
Group (HC or GD)					−0.03 (0.01)	−2.29	0.024
Risk sensitivity					0.03 (0.01)	4.44	<0.001
***Model 3***	−342	0.216	0.023	−3.98			**<0.001**
Group (HC or GD)					−0.03 (0.01)	−2.34	0.021
Risk sensitivity					0.04 (0.01)	4.77	<0.001
Group × Risk sensitivity					−0.03 (0.01)	−2.05	0.042
**b. Modulation of DICI by trait impulsivity**							
***Model 1***	−323	0.080	0.080	12.48			**<0.001**
Group (HC or GD)					−0.05 (0.01)	−3.53	0.001
***Model 2***	−325	0.105	0.025	−4.16			**<0.001**
Group (HC or GD)					−0.07 (0.02)	−3.92	0.024
BIS-11					0.02 (0.01)	1.97	0.050
***Model 3***	−330	0.147	0.042	−0.21			**<0.001**
Group (HC or GD)					−0.09 (0.02)	−4.59	<0.001
BIS-11					0.05 (0.02)	3.32	0.001
Group × BIS-11					−0.05 (0.02)	−2.64	0.009

*Note*: Hierarchical regression models testing the modulation of dynamic impulse control index (DICI). Risk sensitivity modulation: Model 1 = Group; Model 2 = Model 1 + Risk sensitivity; Model 3 = Model 2 + Interaction term. Trait impulsivity modulation: Model 1 = Group; Model 2 = Model 1 + BIS-11; Model 3 = Model 2 + Interaction term. Abbreviations: DICI, dynamic impulse control index; GD, gambling disorders; HC, healthy controls; BIS-11, Barratt Impulsiveness Scale version 11.

The bolded *P* values in Table 3 indicate the overall hierarchical regression model is statistically significant, showing the set of predictors in each model significantly explains variance in dynamic impulse control (DICI).

Given the significant interaction (*P* = 0.042), simple slope analyses revealed that while both groups exhibited positive risk sensitivity*–*DICI associations (HC: *β* = 0.040 ± 0.011, *t*(61) = 3.458, *P* = 0.001; GD: *β* = 0.013 ± 0.006, *t*(82) = 2.205, *P* = 0.030), the effect was stronger in HCs than in patients with GD (*t*(90.57) = 2.083, *P* = 0.040, *d* = 0.338; Figure 2c).

### Trait impulsivity modulates dynamic impulse control

As shown in Table 3b (modulation by trait impulsivity), the baseline model (Model 1: Group-only, identical to Table 3) remained significant (*R* ^2^ = 0.080, *F* = 12.48, *P* < 0.001). Adding BIS-11 scores (Model 2) significantly enhanced prediction (Δ*R* ^2^ = 0.025, Δ*F* = −4.16, *P* < 0.001). The BIS-11 × Group interaction (Model 3) further increased explanatory power (Δ*R* ^2^ = 0.042, Δ*F* = −0.21, *P* < 0.001), demonstrating unique variance contribution.

For the significant interaction (*P* = 0.009), simple slope analyses indicated that trait impulsivity modulated DICI exclusively in HCs (*β* = 0.050 ± 0.021, *t*(61) = 2.403, *P* = 0.019), with no effect in GD patients (*β* = 0.0001 ± 0.0065, *t*(82) = 0.009, *P* = 0.993) (Figure 2d). This suggests intact trait impulsivity-dependent control only in healthy individuals.

## Discussion

This study demonstrates a significant impairment in dynamic impulse control among patients with GD during escalating risk–reward scenarios, as quantified by attenuated coupling between cumulative rewards and RTs for risky ‘continue’ choices. Unlike HCs, who normatively lengthen deliberation times as stakes rise, patients with GD failed to increase deliberation time as stakes rose, providing a mechanistic explanation for persistent risk-taking despite escalating losses, a hallmark of GD pathophysiology. This attenuated DICI was computationally linked to significantly reduced risk sensitivity, indicating an impaired ability to subjectively weigh escalating threat magnitudes, while outcome-based measures and risk preference remained intact. Consequently, the attenuated DICI in GD reflects a disruption in contextual risk evaluation that undermines the engagement of inhibitory resources under mounting stakes. Thus, the DICI deficit in patients with GD points to a specific impairment in the dynamic regulation of impulse control amid escalating risk, a mechanistic dysfunction that may underpin real-world gambling persistence independent of overall task performance outcomes. Notably, both groups accrued less than the theoretically optimal earnings (200 Renminbi), but the GD group’s failure to adapt deliberation to stakes suggests a suboptimal process that could exacerbate losses in prolonged gambling.

While we interpret the attenuated DICI primarily as an index of impaired dynamic impulse control, this metric likely reflects the contribution of broader computational processes. Specifically, the escalating stakes in our task inherently increase decision conflict, the competition between the urge to continue (driven by potential reward), and the urge to stop (motivated by escalating risk). This attenuated DICI could thus signify a disruption in the dynamic weighting of evidence under conflict, where patients accumulate less cautionary evidence against continuing, rather than a deficit in pure inhibitory control per se. This interpretation aligns with computational frameworks like the drift-diffusion model, where reduced response caution under conflict accounts for impulsive choices. Future studies incorporating trial-by-trial measures of conflict (e.g. via frontal theta oscillations) and formal evidence accumulation models could further dissociate these processes in GD.

Moreover, the relationship between trait impulsivity and dynamic control was fundamentally disrupted in GD. Whereas HCs showed a compensatory pattern where higher trait impulsivity positively modulated DICI strength, this adaptive coupling was absent in GD patients, despite their elevated impulsivity scores. This dissociation suggests a failure in translating motivational states into context-appropriate inhibitory adjustments. Importantly, these deficits refine existing models of GD by highlighting a triadic impairment: (1) compromised dynamic impulse control during action preparation; (2) blunted sensitivity to escalating risk; and (3) decoupling of trait impulsivity from adaptive behavioral regulation. These findings align with known dysfunction in cortico-subcortical ‘braking’ networks (e.g. pre-SMA, IFG) (Clark et al., 2009; Gavazzi et al., 2025; Kaasinen et al., 2023; Sescousse et al., 2013) implicated in context-dependent inhibitory scaling (Lohse et al., 2023; Meder et al., 2016), underscoring the potential for targeted neuromodulation or cognitive remediation focused on risk evaluation.

Several limitations of this study should be acknowledged. The generalizability of our findings may be constrained by the exclusive use of an all-male sample and the deliberate exclusion of comorbidities, which limits the representation of the full clinical heterogeneity inherent in GD and may introduce selection biases, particularly given that GD is a low base-rate disorder often characterized by diverse phenotypic presentations. Furthermore, the absence of study pre-registration sharing represents a methodological limitation. The observed lower variability in DICI and risk sensitivity among patients with GD, which contrasts with the expected heterogeneity in a disorder often described by typological models like the Pathways Model (Lopes & Tavares, 2024; Mestre-Bach et al., 2022), may suggest that our sample captures a specific subtype characterized by consistent deficits in dynamic control and risk evaluation. Consequently, future studies should prioritize the recruitment of larger, phenotypically diverse samples and incorporate targeted subtyping measures to clarify whether individuals exhibiting this cognitive profile represent a distinct etiological pathway within the broader GD population.

In conclusion, our results provide compelling evidence that the core pathology of GD involves a profound, context-dependent failure to dynamically regulate impulses in response to escalating risk and reward contingencies, driven by impaired risk evaluation and a decoupling from underlying impulsivity traits. This framework moves beyond static trait-based models, offering a dynamic, mechanism-focused perspective crucial for developing targeted interventions for GD.

## Supporting information

10.1017/S0033291726104322.sm001 Zhong et al. supplementary material Zhong et al. supplementary material

## Data Availability

Due to ethical considerations and restrictions imposed by participant consent, the raw data cannot be publicly shared. For inquiries regarding data access, please contact M.Z. All data requests will be evaluated on a case-by-case basis. Access to the data requires the submission of a detailed research proposal and approval from a relevant ethics committee.

## Supplementary material

The supplementary material for this article can be found at http://doi.org/10.1017/S0033291726104322.
