# Supplementary material for: Attenuated dynamic impulse control in risky action under the escalating risk and reward in gambling disorder
Source: Psychol Med. 2026 May 11;56:e141. doi: 10.1017/S0033291726104322 (PMC13161805; doi:10.1017/S0033291726104322)
Supplement: Zhong et al. supplementary material [file S0033291726104322sup001.docx]

**Supplementary Methods**

**Clinical diagnoses and sample characterization**

GD diagnoses were independently verified by certified psychiatrists specializing in addiction psychiatry at the Shanghai Mental Health Center, all of whom held advanced training in DSM-5 criteria (American Psychiatric Association, 2013) and had extensive clinical experience in diagnosing GD. Diagnoses of GD were verified using the Chinese translated version of the Structured Clinical Interview for DSM-5 Disorders—Clinician Version (SCID-5-CV) (First, Williams, Karg, & Spitzer, 2016), a well-validated, standardized tool that contains dedicated modules for GD assessment. This version was translated under the leadership of Professor Lipeng Fei from the Shanghai Mental Health Center, with contextual adaptations for Chinese participants. In Shanghai, online gambling is highly accessible due to digital infrastructure, with common forms including live-dealer casino games, sports betting, and slot-machine simulations (Zhong, Du, & Zhao, 2024). Among our GD sample, 81% (n=67) reported primary engagement with online platforms, while 19% (n=16) preferred offline settings (e.g., card games). Inpatients (n=21) received intensive residential care with an average treatment duration of 1.2±0.4 months. All inpatients were undergoing first-line therapy focused on impulse control and relapse prevention. Outpatient (n=62) treatment data were incomplete due to the cross-sectional focus on baseline status, non-standardized records across clinics, and stigma-related non-disclosure.

GD diagnoses were independently verified by certified psychiatrists specializing in addiction psychiatry at the Shanghai Mental Health Center, all of whom held advanced training in DSM-5 criteria (American Psychiatric Association, 2013) and had extensive clinical experience in diagnosing GD. Diagnoses of GD were verified using the Chinese translated version of the Structured Clinical Interview for DSM-5 Disorders—Clinician Version (SCID-5-CV) (First, 2020), a well-validated, standardized tool that contains dedicated modules for GD assessment. This version was translated under the leadership of Professor Lipeng Fei from the Shanghai Mental Health Center, with contextual adaptations for Chinese participants. In Shanghai, online gambling is highly accessible due to digital infrastructure, with common forms including live-dealer casino games, sports betting, and slot-machine simulations (Zhong, Du, & Zhao, 2024). Among our GD sample, 81% (n=67) reported primary engagement with online platforms, while 19% (n=16) preferred offline settings (e.g., card games). Inpatients (n=21) received intensive residential care with an average treatment duration of 1.2±0.4 months. All Inpatients were undergoing first-line therapy focused on impulse control and relapse prevention. Outpatient (n=62) treatment data were incomplete due to: 1) cross-sectional focus on baseline status; 2) non-standardized records across clinics; 3) stigma-related non-disclosure.

**Supplementary Tables**

**Supplementary Table S1. Specifications of Hierarchical Bayesian models.**

| **Component** | **Model 1: Logistic Regression**  **(Risk Sensitivity & Preference)** | **Model 2: Linear Regression**  **(DICI)** |
| --- | --- | --- |
| Response variable | Binary choice: continue (1) vs. stop (0) | Log-transformed reaction time (log(RT)) for "continue" decisions |
| Predictors | Cumulative reward sum ($\Sigma_{t}$) | Round number (*r*), within-round decision number (*t*), cumulative reward sum ($\Sigma_{t}$), choice conflict ($\mathrm{cc}_{t}$) |
| Mathematical form | ${p(continue)}_{t}=\frac{1}{1+exp(-(\beta_{0}+\beta_{1}\Sigma_{t})}$ | *log* (*RT*) = $\gamma_{0}$+$\gamma_{1}$*r* +$\gamma_{2}$*t* +$\gamma_{3}\Sigma_{t}$ + $\gamma_{4}\mathrm{cc}_{t}$ |
| Indiv.-level parameters | $\beta_{0}$(intercept), $\beta_{1}$(slope for $\Sigma_{t}$) | $\gamma_{0}$ (intercept), $\gamma_{1}$, $\gamma_{2}$, $\gamma_{3}$, $\gamma_{4}$ (slopes) |
| Indiv.-level prior | $\beta_{0}$∼*N* ($\mu_{\beta_{0}}$, $\sigma_{\beta_{0}}$)  $\beta_{1}$∼*N* ($\mu_{\beta_{1}}$, $\sigma_{\beta_{1}}$) | $\gamma_{i}$∼*N* ($\mu_{\gamma_{i}}$, $\sigma_{\gamma_{i}}$) for *i* = 0, 1, ..., 4 |
| Group-level hyperpriors | $\mu_{\beta_{0}}$~*Cauchy* (0,5); $\sigma_{\beta_{0}}$~*Half-Cauchy* (0,1)  $\mu_{\beta_{1}}$~*Cauchy* (0,1); $\sigma_{\beta_{1}}$~*Half-Cauchy* (0,0.5) | $\mu_{\gamma_{0}}$~*Cauchy* (0,10); $\sigma_{\gamma_{0}}$~*Half-Cauchy* (0,5)  $\mu_{\gamma_{i}}$~*Cauchy* (0,5); $\sigma_{\gamma_{i}}$~*Half-Cauchy* (0,5) for i>0 |
| Derived measures | Risk sensitivity = $\beta_{1}$  Risk preference = -$\beta_{0}$/$\beta_{1}$ | Dynamic Impulse Control Index (DICI) = γ₃ (slope for $\Sigma_{t}$) |
| Implementation (Stan) | 4 chains, 8000 iterations (87.5% warm-up) | 2 chains, 2000 iterations (50% warm-up) |
| Convergence Diagnostics | R-hat < 1.05, <1% divergent transitions, <1% max tree depth | R-hat < 1.05, <1% divergent transitions, <1% max tree depth |

*Note.* DICI, Dynamic Impulse Control Index; Indiv., Individual.

**Supplementary Figures**

**Supplementary Figure 1.** **Hierarchical Bayesian regression models demonstrated adequate fit (R-square reached up to ~0.6). (a)** Bayesian R-squared for the Hierarchical Bayesian linear model in healthy controls (HCs). **(b)** Bayesian R-squared for the Hierarchical Bayesian linear model in patients with gambling disorder (GD). **(c)** Bayesian R-squared for the Hierarchical Bayesian logistic model in HCs. **(d)** Bayesian R-squared for the Hierarchical Bayesian logistic model in patients with GD. Abbreviations: HC, healthy controls; GD, gambling disorder.

**Reference**

First, M. B., Williams, J.B.W., Karg, R.S., & Spitzer, R.L. (2020). *Structured Clinical Interview for DSM-5 Disorders Research Version (SCID-5-RV)* (M. R. Philips, Trans. M. R. Philips Ed.): Peking University Press.

American Psychiatric Association (Ed.) (2013). *Diagnostic and Statistical Manual of Mental Disorders 5th edn*.

First, M. B., Williams, J. B. W., Karg, R. S., & Spitzer, R. L. (2016). *User's guide for the SCID-5-CV Structured Clinical Interview for DSM-5® disorders: Clinical version*. Arlington, VA, US: American Psychiatric Publishing, Inc.

Zhong, G., Du, J., & Zhao, M. (2024). Understanding and addressing the challenges of online gambling addiction in mainland China. *General Psychiatry, 37*(2), e101477. doi:10.1136/gpsych-2023-101477
